# Effect of 5-fluorouracil on *Pseudomonas aeruginosa*: impact on virulence, biofilm formation, and bacterial growth

**DOI:** 10.3389/fmicb.2025.1584479

**Published:** 2025-07-18

**Authors:** Amani A. Niazy, May M. Alrashed, Abdurahman A. Niazy

**Affiliations:** ^1^Department of Clinical Laboratory Sciences, College of Applied Medical Sciences, King Saud University, Riyadh, Saudi Arabia; ^2^Department of Oral Medicine and Diagnostic Science, College of Dentistry, King Saud University, Riyadh, Saudi Arabia

**Keywords:** *Pseudomonas aeruginosa*, 5-fluorouracil, antimicrobial resistance, chemotherapeutic agent, opportunistic pathogen, biofilm

## Abstract

*Pseudomonas aeruginosa* is a Gram-negative opportunistic pathogen that poses a significant threat to public health due to its inherent and acquired resistance to multiple antibiotics. This mini-review explores the antimicrobial potential of 5-fluorouracil (5-FU), a chemotherapeutic agent normally used in oncology, and currently under investigation for its bacteriostatic and antibiofilm effects against *P. aeruginosa*. 5-FU functions by inhibiting thymidylate synthase, disrupting nucleotide metabolism, and interfering with essential bacterial processes, such as quorum sensing. Studies *in vitro* have demonstrated its ability to inhibit biofilm creation and decrease virulence, but findings about its impact on preformed biofilms have been contradictory. Synergistic interactions between 5-FU and antibiotics, especially gentamicin, have shown promise in enhancing antibacterial effectiveness. The aim of this mini-review was to consolidate current findings, pinpoint research gaps, and suggest future directions for potentially repurposing 5-FU as an adjunctive treatment for *P. aeruginosa*. By placing the current evidence in context, we hope to guide further studies toward determining the clinical viability of 5-FU as a treatment option against this formidable pathogen.

## 1 Introduction

*Pseudomonas aeruginosa* is a ubiquitous Gram-negative bacterium that is found in the environment and is often associated with a variety of life-threatening infections, particularly in individuals who are immunocompromised (Moradali et al., 2017). The organism is highly adaptable and is commonly found in chronic infections, infections associated with diabetes, and burn-wound infections. Its adaptability to various environments and capacity to form biofilms enhance its adherence to wounds. Furthermore, it secretes enzymes and toxins that damage tissue and immune cells, which lead to severe inflammation and delayed healing of wounds (Phan et al., 2023).

*P. aeruginosa* also uses a combination of intrinsic mechanisms, such as efflux pumps, reduced permeability, acquired resistance through mutations and horizontal gene transfer, and adaptive strategies, such as biofilm formation and quorum sensing, which enhance its resilience and decrease the effectiveness of treatments (Moradali et al., 2017). The World Health Organization (WHO) classifies *P. aeruginosa* as a pathogen of critical priority due to its high level of resistance to multiple antibiotics, including carbapenems and cephalosporins (WHO, 2017). This crisis of antibiotic resistance underscores the need for innovative strategies to manage *P. aeruginosa* infections.

One option is the repurposing of established drugs, such as 5-fluorouracil (5-FU). 5-FU was first introduced in the late 1950s and is a pyrimidine analog that is noted for its success in treating solid tumors (Heidelberger et al., 1957; Shirasaka, 2009). After decades of primary use in oncology, emerging evidence suggests that 5-FU also exhibits antimicrobial activities against a range of bacterial species (Sedlmayer et al., 2021; Soo et al., 2016). The aim of this mini-review was to delve deeper into the understanding of how 5-FU impacts *P. aeruginosa* in particular and to assess its potential as a treatment option. To this end, we reviewed relevant studies, summarized the findings, and evaluated whether additional research into 5-FU's antimicrobial use is justified.

## 2 Pseudomonas aeruginosa: an opportunistic pathogen

### 2.1 General characteristics

*P. aeruginosa* is a rod-shaped, motile, Gram-negative bacterium with polar flagella. Due to its minimal nutritional requirements, it thrives in diverse ecological niches, which range from soil and water to plant surfaces (Wu and Li, 2015). The Centers for Disease Control and Prevention (CDC) have listed *P. aeruginosa* as one of the key organisms that cause healthcare-associated infections (CDC, n.d.). In 2017, it was estimated that multidrug-resistant *P. aeruginosa* caused approximately 32,600 infections among hospitalized patients in the United States, as well as around 2,700 deaths (CDC, 2021). Furthermore, the CDC identified multidrug-resistant *P. aeruginosa* as one of the top 6 most alarming antibiotic resistance threats in the U.S. in 2021 (CDC, 2021).

The genome of *P. aeruginosa* is significantly larger than that of most other sequenced bacteria and ranges between 5.5 and 7 Mbp. The genome of the reference strain PAO1 contains over 500 regulatory genes alone, which contribute to its exceptional adaptability. This extensive genetic repertoire amplifies its ability to mutate, adapt, and resist the effects of antibiotic treatments (Diggle and Whiteley, 2020). *P. aeruginosa* poses problems in hospital settings due to its ability to develop resistance to multiple classes of antibiotics, which sometimes even occurs in the middle of treatment (Lister et al., 2009).

### 2.2 Virulence factors and antibiotic resistance

*P. aeruginosa* exhibits diverse virulence factors that play an essential role in the organism's pathogenesis. These include efflux pumps (Li et al., 2015), flagella, type IV pili, biofilm formation (Taylor et al., 2014), the secretion of alkaline protease, elastase, exotoxin A, and pigments such as pyoverdine and pyocyanin (Breidenstein et al., 2012; Jurado-Martín et al., 2021). Coupled with acquired antibiotic resistance, these factors are regulated by incredibly complex interconnected pathways and signaling systems. This extensive regulation grants the pathogen a remarkable ability to adapt to various environments. Research in this field remains active, and efforts are being made to decipher the mechanisms behind resistance and adaptability in this pathogen.

## 3 5-Fluorouracil (5-FU)

5-FU is a fluorinated analog of uracil that primarily inhibits the enzyme thymidylate synthase (TS), which leads to the depletion of thymidylate and subsequent inhibition of both DNA and RNA synthesis. In cancer cells, 5-FU is metabolically activated and changes into fluorodeoxyuridine monophosphate (FdUMP), which forms a complex with TS and folate cofactors. This ternary complex inhibits the transformation of deoxyuridine monophosphate (dUMP) to thymidylate (dTMP), resulting in impaired DNA replication and thus cancer-cell death (Longley et al., 2003; Wilson et al., 2014).

Additionally, 5-FU can be incorporated into RNA as fluorouridine triphosphate (FUTP), which disrupts RNA function and protein synthesis (Scartozzi et al., 2011). 5-FU was first synthesized by Heidelberger et al. (1957). It has been in clinical use for almost 60 years and remains one of the most widely used chemotherapeutic agents. It displays broad-spectrum activity against many solid tumors, including colorectal, pancreas, breast, head, and neck tumors (Wilson et al., 2014).

### 3.1 Known antimicrobial activities

Beyond its antineoplastic properties, 5-FU has exhibited antimicrobial effects against both Gram-positive and Gram-negative organisms. Numerous studies *in vitro* report the growth inhibition of both types of bacteria, suggesting that the cytotoxic mechanisms employed against tumor cells can also interfere with bacterial nucleotide metabolism (Attila et al., 2009; García-Contreras et al., 2013; Sedlmayer et al., 2021). This may be attributed to the similarity between mammalian and bacterial TS enzymes (Islam et al., 2018). Therefore, repurposing 5-FU to block DNA synthesis in replicating organisms is an attractive new approach to combat problematic organisms such as *P. aeruginosa* (Quezada et al., 2020; Soo et al., 2016). However, the relevant studies have predominantly been *in vitro* studies.

## 4 Effects of 5-FU on *P. aeruginosa*

### 4.1 Growth

Di Bonaventura et al. (2022) investigated the bacteriostatic effects of 5-FU on *P. aeruginosa* strains isolated from patients with cystic fibrosis and observed minimal inhibitory concentrations (MICs) ranging from 128 to >1,024 μg/mL. Patil et al. (2023) reported that the MIC of 5-FU against *P. aeruginosa* was 256 μg/mL. Moreover, 5-FU has been shown to impede the growth of *P. aeruginosa* in a dose-dependent manner and achieves 100% inhibition at concentrations of 25 μg/mL and above (Niazy et al., 2024).

### 4.2 Biofilms

5-FU significantly inhibits biofilm formation of *P. aeruginosa*. One study has reported a threefold reduction in biofilm formation of *P. aeruginosa* PA14 at a 5-FU concentration of 25 mM in lysogeny broth (LB) medium, as well as a 33-fold reduction at 200 mM. In M9 glucose medium, the same research indicated a 56% reduction in biofilm formation with 10 mM 5-FU (Ueda et al., 2009). A more recent study identified 5-FU as a potential anti-biofilm agent against *P. aeruginosa* strains that relevant to cystic fibrosis, which was determined using the RP73 strain, a widely employed benchmark in chronic illness studies. This work reported that 5-FU could significantly inhibit biofilm formation and suppress quorum-sensing-regulated virulence factors in *P. aeruginosa* (Di Bonaventura et al., 2023).

Another investigation demonstrated that 5-FU effectively inhibited *P. aeruginosa* biofilm formation in a dose-dependent manner. The findings revealed reductions of 58% in biofilm biomass at 0.1 μg/mL, 63% at 0.5 μg/mL, and over 70% at concentrations of 12 and 100 μg/mL (Niazy et al., 2024). However, when assessing the dispersal and killing activities of 5-FU against 24-h-old biofilms, the results showed that the drug actually stimulated the mature biofilm biomass rather than reducing it (Di Bonaventura et al., 2022). This was also reported in another study, which specifically stated that at concentrations of 12 and 100 μg/mL, 5-FU caused an increase in the 48-h pre-formed biofilm biomass by 129.10 and 164.86% compared to the untreated sample, respectively (Niazy et al., 2024).

### 4.3 Other virulence factors

At subinhibitory concentrations, 5-FU significantly upregulates the quorum-sensing gene (*lasI*) and various efflux pump genes (*mexA, mexB*, and *mexC*), but it downregulates the alkaline protease gene (*aprA*) in *P. aeruginosa* PaPh32. The exotoxin (*toxA*) and alginate (*algD*) genes remain unaffected, suggesting that 5-FU selectively modifies key virulence pathways, particularly quorum-sensing and efflux mechanisms (Di Bonaventura et al., 2022). Another study assessed the impact of 5-FU on the virulence factors elastase, pyocyanin, and alkaline protease in *P. aeruginosa* strains PAO1 and PA14 and some clinical isolates. These findings suggested that while 5-FU is generally effective in reducing virulence factors, its efficacy is not consistent across all clinical strains, and in some situations, it might even elevate the production of certain virulence factors such as pyocyanin (García-Contreras et al., 2013).

Furthermore, Kirienko et al. (2016) demonstrated that 5-FU can reduce pyoverdine biosynthesis, thereby potentially attenuating the bacterium's virulence. 5-Fluorouracil also shows a significant suppressive effect on quorum-sensing QS-regulated phenotypes in *P. aeruginosa*, including decreased elastase activity, pyocyanin and rhamnolipid production, and swarming motility. Additionally, 5-FU significantly attenuates the virulence of *P. aeruginosa*, as evidenced by its decreased pathogenic impact in the barley germination assay (Ueda et al., 2009).

### 4.4 Potential synergistic effects

**Figure 1:**
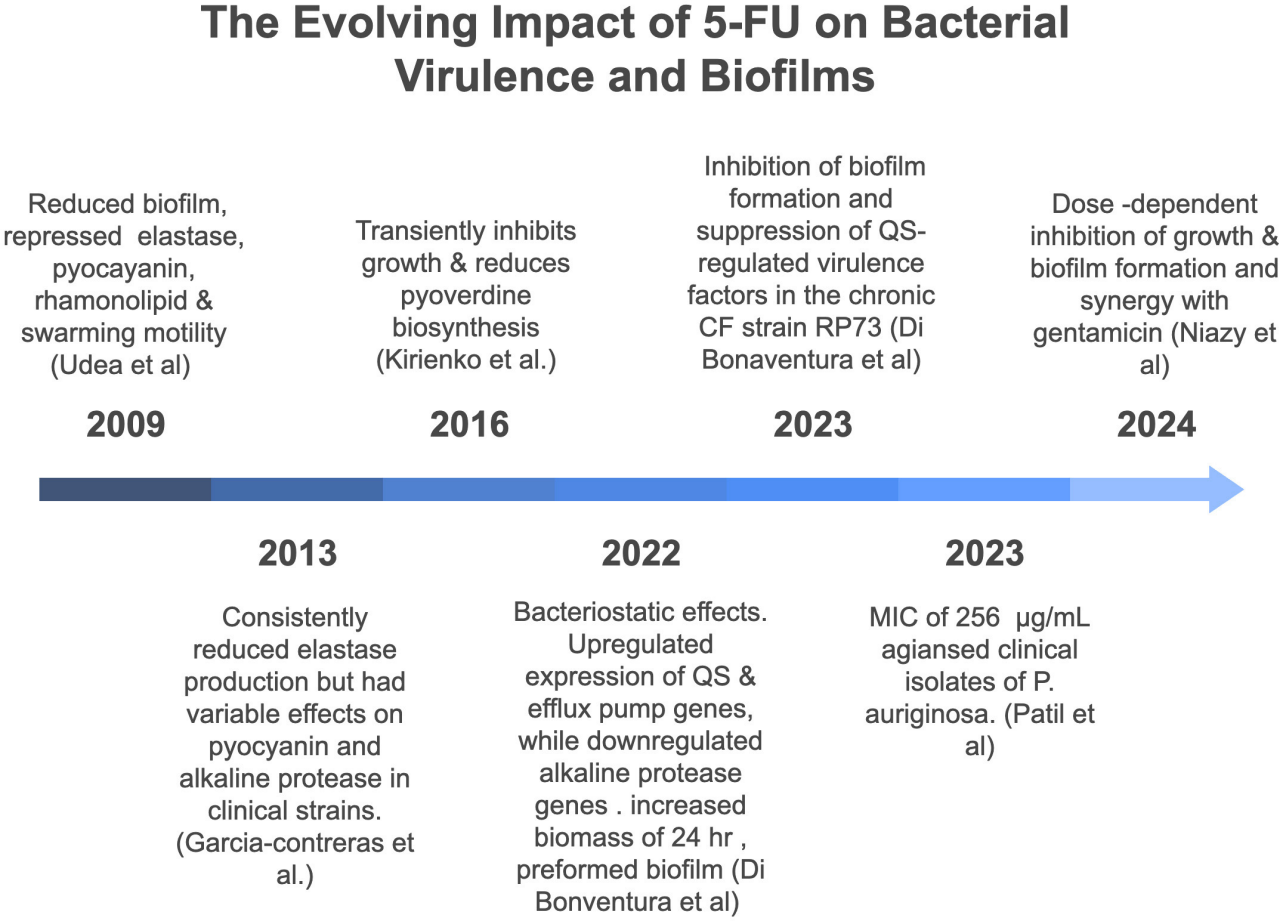
Chronological overview of key studies investigating the effects of 5-fluorouracil (5-FU) on virulence and biofilm formation of *Pseudomonas aeruginosa* (2009–2024).

Investigations into the synergistic effects of 5-FU with antibiotics are rare and have not been extensively explored. In one particular study, the combination of 5-FU and gentamicin demonstrated strong synergy, and the fractional inhibitory concentration index (FICI) was 0.31 for growth inhibition, while a ZIP synergy score of approximately 14.2 was obtained for biofilm inhibition, which suggests potential synergy. In contrast, the combination of 5-FU and meropenem presented an additive effect with an FICI of 0.56 for growth inhibition. However, the ZIP synergy score for biofilm inhibition was 0.97, indicating no synergy (Niazy et al., 2024). Figure 1 summarizes key findings on the effects of 5-FU on *P. aeruginosa* virulence and biofilm formation reported between 2009 and 2024.

## 5 Challenges and considerations

Repurposing 5-FU for infectious disease treatment presents a significant challenge due to its toxicity profile in humans. Adverse effects can occur when 5-FU is systemically administered at doses commonly used in cancer therapy, such as myelosuppression and gastrointestinal toxicity (Grem et al., 2001). Safely optimizing 5-FU as an antibacterial agent requires a multifactorial approach—balancing efficacy against *P. aeruginosa* with strategies to minimize toxicity. This can be achieved not only through dose adjustment but also by selecting alternative routes of administration. For example, topical 5-FU formulations (e.g., 5% creams used for actinic keratosis and superficial basal-cell carcinoma) have demonstrated minimal systemic absorption (Levy et al., 2001; Ceilley, 2012) Inhalation via nebulizers has also shown promise, achieving high pulmonary drug concentrations with minimal systemic exposure in both animal models and clinical settings (Hitzman et al., 2006; Okuda and Okamoto, 2020). Ultimately, achieving a balance between antimicrobial efficacy and acceptable toxicity will be essential for the successful repurposing of 5-FU in infectious disease contexts.

Many Gram-negative bacteria, including *P. aeruginosa*, have outer-membrane permeability barriers and robust efflux-pump systems that can limit the intracellular accumulation of 5-FU (Lorusso et al., 2022). Bacteria can also evolve alternative metabolic pathways or acquire mutations in target enzymes, such as TS, to bypass the inhibitory effects of 5-FU (Choi et al., 2016). Thorough understanding of these resistance mechanisms is crucial for developing effective strategies for the use of 5-FU in infection treatment. This is particularly important as it has been observed that while 5-FU can deter the initial formation of *P. aeruginosa* biofilms, it can also enhance the biomass of pre-existing biofilms.

## 6 Future perspectives

### 6.1 Formulation strategies

Ignoring 5-FU as a treatment option may lead to missed opportunities in specific scenarios, such as the use of a high initial dose for the treatment of severe life-threatening infections or as a topical antibiotic for burns or wounds (McKenzie, 2011; Paterson et al., 2016). Additionally, research could focus on innovative drug delivery methods such as liposomal or nanoparticle-based formulations to enhance the local concentration of 5-FU at infection sites while minimizing systemic toxicity (Gao et al., 2018). Localized administration methods, such as inhalation therapies for CF patients and other critically ill patients, could harness 5-FU's antimicrobial properties while reducing off-target effects (Dalhoff, 2014; Drobnic et al., 2005; Somayaji and Parkins, 2015; Wood et al., 2002). The use of modified forms of 5-FU presents another potential approach for utilizing the drug (Patil et al., 2023). With further investigation, these strategies could help to explore the potential of 5-FU as an antibacterial agent.

### 6.2 Combination therapies

Combination therapies targeting distinct bacterial pathways are often used to combat multi-drug-resistant strains. When both DNA replication and cell wall synthesis or protein translation are targeted, bacteria may face multiple lethal hits. This can potentially slow the emergence of resistant mutants (Bognár et al., 2024; Xiao et al., 2023). The inhibition of TS by 5-FU could potentially create synergistic effects when combined with conventional antibiotics such as aminoglycosides. It is essential to test for synergy in both planktonic and biofilm states to determine whether 5-FU can help overcome drug resistance or enable the use of reduced antibiotic doses, thereby decreasing toxicity. For example, 5-FU could serve as an initial “helper” agent by weakening bacterial defenses and enhancing the antibiotic's ability to effectively clear the infection (Tamma et al., 2012).

### 6.3 Validation *in vivo*

Translational research involving animal models of infection by *P. aeruginosa* is essential to confirm the observations *in vitro*. For instance, mouse-wound models have proven to be a valuable tool in demonstrating the efficacy of antimicrobial biomaterials for infection control and wound healing. Studies have shown that pH-responsive hydrogels loaded with antimicrobial agents can reduce bacterial loads and enhance the closure of infected wounds. These biomaterials respond to changes in the wound environment (such as acidity) to release antimicrobial compounds precisely where needed, which improves bacterial clearance and accelerates tissue regeneration (Monroe and Fikhman, 2023).

Another example involves a mouse model of peritonitis *in vivo*, in which Stenger et al. (2015) used thioridazine (an antipsychotic medication) as a helper compound to enhance the effects of dicloxacillin against methicillin-resistant Staphylococcus aureus (MRSA). They concluded that the combination is effective, and thioridazine could enhance the activity of dicloxacillin against MRSA. Such studies could offer valuable insights into how 5-FU affects infections in different anatomical sites within the host and how the host responds to its administration. This approach could help to determine whether 5-FU is a viable therapeutic option, improve our understanding of its efficacy, safety, and mechanisms of action *in vivo*, and pave the way for well-informed clinical applications.

## 7 Conclusion

**Figure 2:**
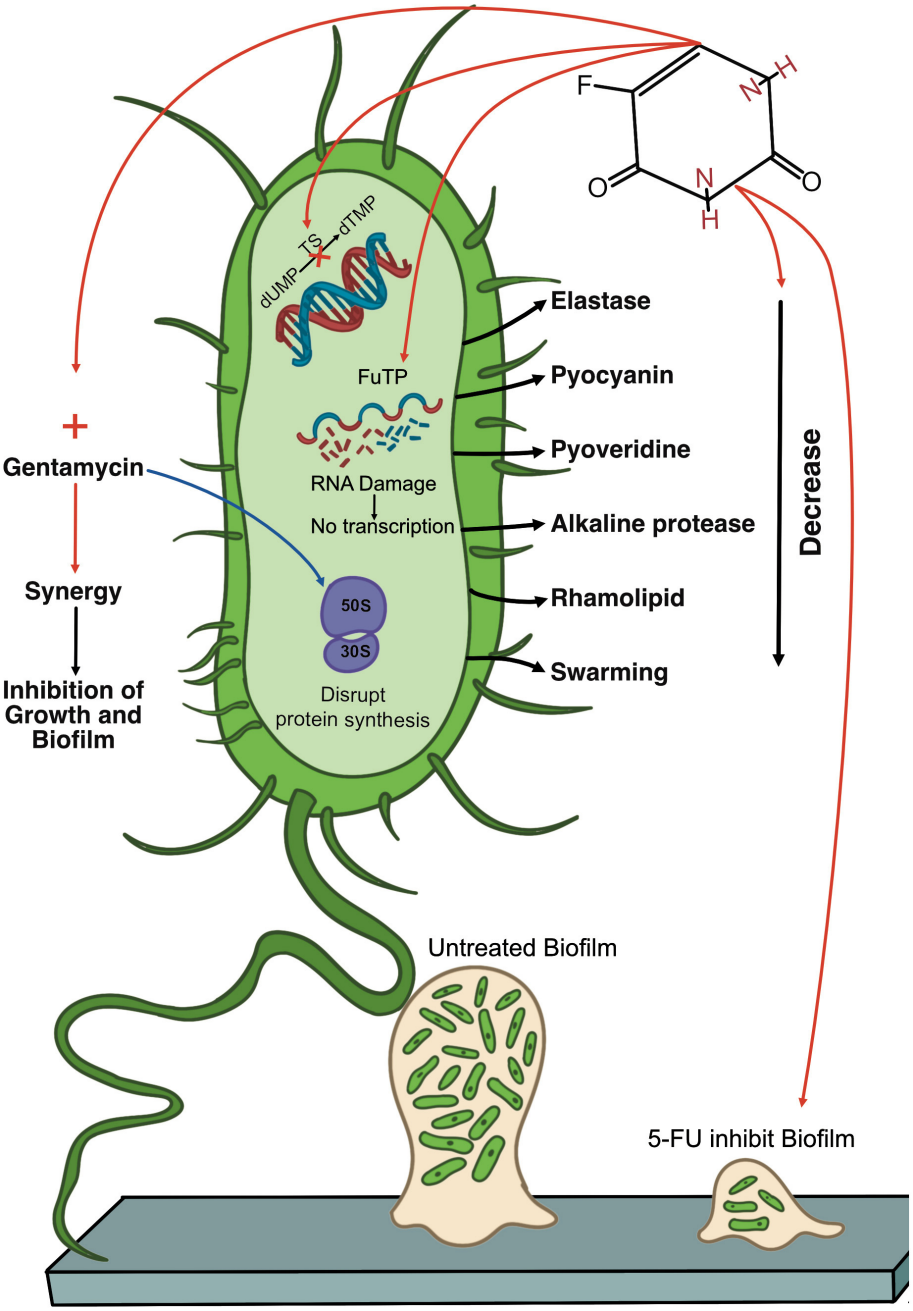
Diagram of the multifaceted antimicrobial activity of 5-fluorouracil (**5-FU**) against *Pseudomonas aeruginosa*. The diagram shows a single *P. aeruginosa* cell (green) and highlights key virulence factors (e.g., elastase, pyocyanin, pyoverdine, alkaline protease, and rhamnolipid), as well as the bacterium's surface appendages (pili and flagella). Inside the cell, 5-FU is metabolized into fluorouracil triphosphate (**FUTP**), which can be aberrantly incorporated into RNA, where it disrupts normal transcription and protein production. Additionally, 5-FU inhibits thymidylate synthase (**TS**), thereby inhibiting DNA synthesis. 5-FU also reduces virulence factor production, which reduces levels of elastase, pyocyanin, pyoverdine, alkaline protease, and rhamnolipid. 5-FU also shows potential synergy with gentamicin (red arrow), resulting in enhanced inhibition of bacterial growth and biofilm formation. The figure contrasts an untreated biofilm **(left)** with a biofilm exposed to 5-FU **(right)**, illustrating that 5-FU can block early-stage biofilm establishment. However, its impact on preformed biofilms can be complex, with some studies indicating increased biomass. 5-FU, 5-fluorouracil; FUTP, fluorouracil triphosphate; TS, thymidylate synthase; dTMP, thymidine monophosphate.

*P. aeruginosa* is a formidable pathogen and a significant threat to public health, which emphasizes the urgent need for new treatment options. However, the development of novel antibiotics is a lengthy and resource-intensive process. A major advantage of drug repurposing is its capacity to significantly reduce the time required to obtain new treatments, thus making them more readily accessible to address unmet clinical needs. Despite its known toxic effects, 5-FU has been clinically utilized for decades in cancer treatment and has demonstrated inhibitory effects *in vitro* against *P. aeruginosa*, including suppression of growth and biofilm formation. However, current findings remain preliminary and underscore the complex and multifaceted interactions between 5-FU and *P. aeruginosa*. Figure 2 illustrates the effects of 5-FU on *P. aeruginosa* as reported in previous studies.

Gene expression studies reveal that 5-FU exerts intricate effects on *P. aeruginosa* virulence, upregulating genes associated with efflux pumps and quorum-sensing while downregulating others, such as alkaline protease. However, such gene expression changes may not result from a direct regulatory interaction with 5-FU. Instead, bacteria often activate efflux and QS pathways as part of a generalized stress response. In *P. aeruginosa*, exposure to oxidative, nitrosative, or chemical stress can induce these systems to enhance survival. Such responses can be secondary effects, reflecting the cell's adaptation to environmental stressors rather than specific gene targeting by the compound (Lee and Zhang, 2015; Muller et al., 2007).

Notably, 5-FU can inhibit initial biofilm formation, yet may increase the biomass of established biofilms, highlighting the complexity of its biofilm-modulating properties. This observation may stem from the timing of drug exposure. When added prior to biofilm establishment, 5-FU likely disrupts initial attachment and matrix production. However, exposure to mature biofilms may trigger stress responses or QS activation, leading to protective adaptations such as enhanced matrix synthesis or cell lysis-mediated eDNA release. These stress-induced mechanisms are well-documented for other subinhibitory concentrations of antimicrobials and could similarly explain 5-FU's paradoxical effects (Kaplan, 2011; Yu et al., 2018).

Additionally, differences in experimental conditions and strain backgrounds likely contribute to the variability in 5-FU MIC values reported across studies. Clinical isolates, such as the 19 *P. aeruginosa* strains obtained from cystic fibrosis patients and tested by Di Bonaventura et al. (2022), may exhibit higher tolerance compared to the standard lab strain PAO1 used by Patil et al. and Niazy et al. Moreover, the use of different growth media Mueller-Hinton by Di Bonaventura et al. (2022) and Patil et al. (2023) vs. PSMM (a defined medium optimized for *P.aeruginosa*) by Niazy et al. (2024) can further influence drug activity and bacterial responses. The influence of growth media on MIC outcomes has been documented in previous studies (Sörensen et al., 2020). These variations in strains, media, and testing conditions represent a key limitation when comparing across *in vitro* studies and may account for conflicting results. This underscore the need for more standardized and comprehensive *in vitro* and *in vivo* investigations to clarify the role of 5-FU.

Despite some conflicting findings, 5-FU has consistently demonstrated inhibitory effects against *P. aeruginosa*, particularly through disruption of biofilm formation and virulence. Its potential as a topical or inhaled antimicrobial, either as monotherapy or in combination with agents like gentamicin, warrants further investigation. To fully assess its therapeutic viability, future research should prioritize (i) *in vitro* and *in vivo* validation using wound and respiratory infection models that mimic clinically relevant delivery routes; (ii) toxicokinetic studies comparing systemic exposure following topical, inhaled, or catheter-lock administration; (iii) combination therapy trials guided by demonstrated *in vitro* synergy; and (iv) laboratory evolution experiments to evaluate resistance development under prolonged sub-MIC exposure. These efforts are essential to clarify 5-FU's antimicrobial mechanisms, optimize dosing strategies, and assess its safety in non-cancer contexts. Collectively, such studies will determine whether 5-FU can be effectively repurposed as a valuable adjunctive or stand-alone treatment option for severe *P. aeruginosa* infections.
